# Ethyl 4-oxo-8-trifluoro­methyl-1,4-dihydro­quinoline-3-carboxyl­ate

**DOI:** 10.1107/S1600536812045321

**Published:** 2012-11-10

**Authors:** B. Garudachari, Arun M. Islor, M. N. Satyanarayan, Thomas Gerber, Eric Hosten, Richard Betz

**Affiliations:** a National Institute of Technology-Karnataka, Department of Chemistry, Organic Chemistry Laboratory, Surathkal, Mangalore 575 025, India; b National Institute of Technology-Karnataka, Department of Physics, Surathkal, Mangalore 575 025, India; c Nelson Mandela Metropolitan University, Summerstrand Campus, Department of Chemistry, University Way, Summerstrand, PO Box 77000, Port Elizabeth, 6031, South Africa

## Abstract

The asymmetric unit of the title compound, C_13_H_10_F_3_NO_3_, contains two independent mol­ecules with similar conformations. In the crystal, N—H⋯O hydrogen bonds link alternating independent mol­ecules into chains in [-110]. In the chain, the quinoline planes of the independent mol­ecules are almost perpendicular to each other, forming a dihedral angle of 89.8 (1)°. π–π inter­actions between the aromatic rings of quinoline bicycles related by inversion centres [for two independent centrosymmetric dimers, the shortest centroid–centroid distances are 3.495 (1) and 3.603 (1) Å] link the hydrogen-bonded chains into layers parallel to (110). Weak C—H⋯F and C—H⋯O inter­actions further consolidate the crystal packing.

## Related literature

For background information about the pharmacological properties of quinoline derivatives, see: Holla *et al.* (2006 ▶); Bekhit *et al.* (2004 ▶); Kaur *et al.* (2010 ▶); Isloor *et al.* (2009 ▶); Vijesh *et al.* (2011 ▶). For graph-set analysis of hydrogen bonds, see: Etter *et al.* (1990 ▶); Bernstein *et al.* (1995 ▶). For the synthesis of the title compound, see: Thomas *et al.* (2011 ▶).
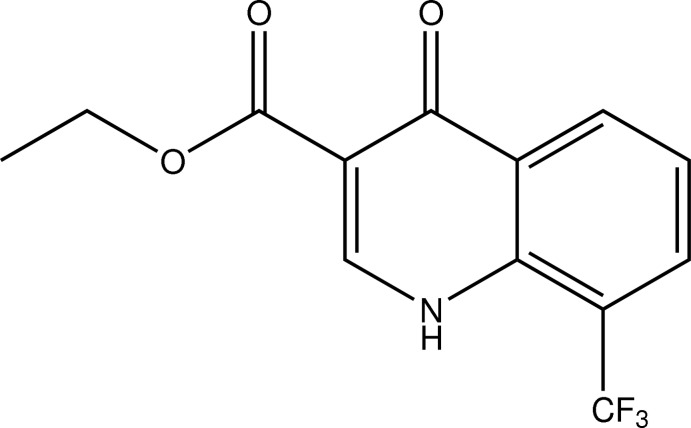


## Experimental

### 

#### Crystal data

C_13_H_10_F_3_NO_3_
*M* *_r_* = 285.22
Triclinic, 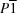

*a* = 9.8248 (3) Å
*b* = 11.0222 (3) Å
*c* = 12.3450 (4) Å
α = 72.934 (1)°
β = 74.167 (1)°
γ = 74.059 (1)°
*V* = 1201.67 (6) Å^3^
*Z* = 4
Mo *K*α radiation
μ = 0.14 mm^−1^
*T* = 200 K
0.53 × 0.38 × 0.32 mm

#### Data collection

Bruker APEXII CCD diffractometer
Absorption correction: multi-scan (*SADABS*; Bruker, 2008 ▶) *T*_min_ = 0.928, *T*_max_ = 0.956
21419 measured reflections
5963 independent reflections
5051 reflections with *I* > 2σ(*I*)
*R*_int_ = 0.016

#### Refinement

*R*[*F* ^2^ > 2σ(*F* ^2^)] = 0.040
*wR*(*F* ^2^) = 0.116
*S* = 1.04
5963 reflections
371 parameters
H atoms treated by a mixture of independent and constrained refinement
Δρ_max_ = 0.39 e Å^−3^
Δρ_min_ = −0.23 e Å^−3^

### 

Data collection: *APEX2* (Bruker, 2010 ▶); cell refinement: *SAINT* (Bruker, 2010 ▶); data reduction: *SAINT*; program(s) used to solve structure: *SHELXS97* (Sheldrick, 2008 ▶); program(s) used to refine structure: *SHELXL97* (Sheldrick, 2008 ▶); molecular graphics: *ORTEP-3* (Farrugia, 2012 ▶) and *Mercury* (Macrae *et al.*, 2008 ▶); software used to prepare material for publication: *SHELXL97* and *PLATON* (Spek, 2009 ▶).

## Supplementary Material

Crystal structure: contains datablock(s) I, global. DOI: 10.1107/S1600536812045321/cv5354sup1.cif

Supplementary material file. DOI: 10.1107/S1600536812045321/cv5354Isup2.cdx

Structure factors: contains datablock(s) I. DOI: 10.1107/S1600536812045321/cv5354Isup3.hkl

Supplementary material file. DOI: 10.1107/S1600536812045321/cv5354Isup4.cml

Additional supplementary materials:  crystallographic information; 3D view; checkCIF report

## Figures and Tables

**Table 1:** Hydrogen-bond geometry (Å, °)

*D*—H⋯*A*	*D*—H	H⋯*A*	*D*⋯*A*	*D*—H⋯*A*
N1—H1⋯O21^i^	0.892 (19)	1.875 (19)	2.6588 (13)	145.5 (16)
N2—H2⋯O11^ii^	0.851 (18)	2.011 (17)	2.7178 (13)	139.9 (16)
N2—H2⋯O12^ii^	0.851 (18)	2.487 (17)	3.0380 (15)	123.2 (14)
C212—H21*B*⋯F22^iii^	0.99	2.46	3.0909 (18)	121
C204—H204⋯O13^iv^	0.95	2.60	3.4691 (18)	153

Symmetry codes: (i) 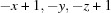; (ii) 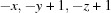; (iii) 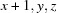; (iv) 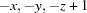.

**Fig. 1.**
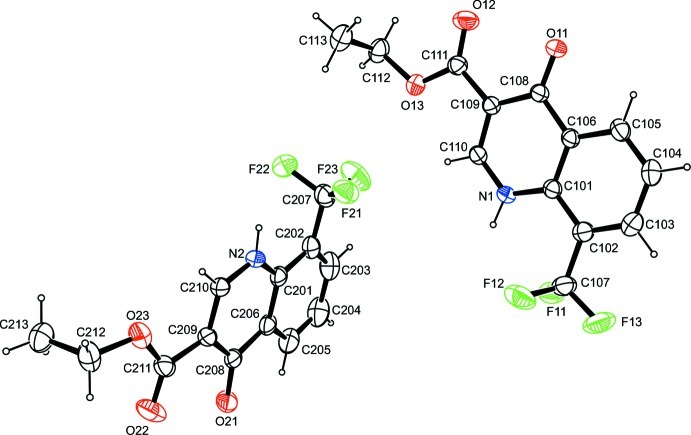
Two independent molecules of the title compound, with atom labels and anisotropic displacement ellipsoids drawn at the 50% probability level.

**Fig. 2.**
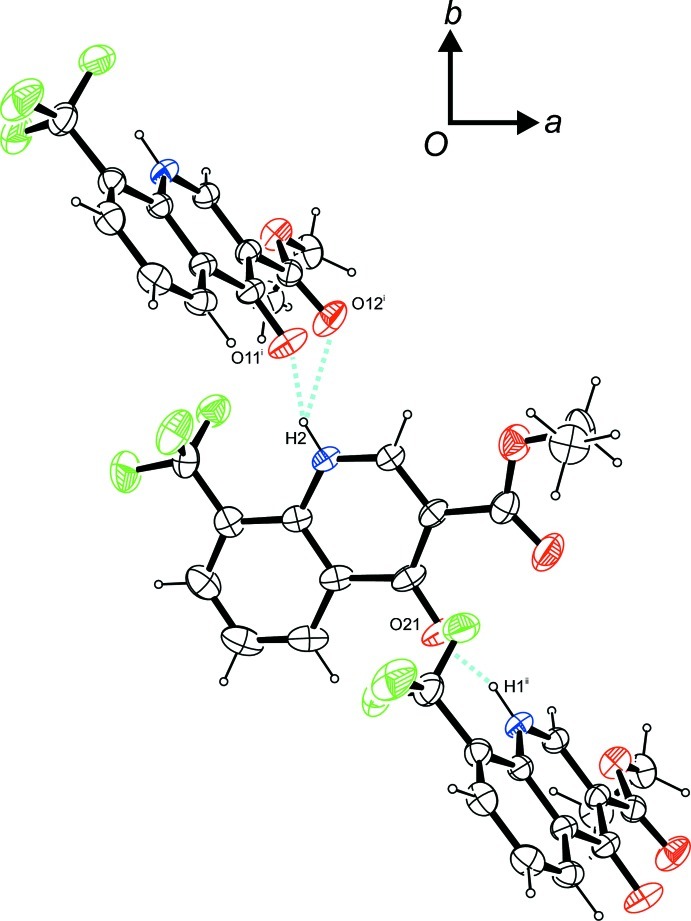
A portion of the crystal packing viewed down the c axis. Dashed lines denote classical N–H···O hydrogen bonds. Symmetry codes: (i) -x, -y + 1, -z + 1; (ii) -x + 1, -y, -z + 1.

## supplementary crystallographic information

### Comment

Quinoline derivatives constitute an important class of compounds that are widely found in plants. A number of synthetic analogues have been developed over the years. Some of them exhibit remarkable effects such as antimicrobial, anti-inflammatory and antimalarial (Holla *et al.*, 2006; Bekhit *et al.*, 2004; Kaur *et al.*, 2010). This follows a broader trend that shows nitrogen-containing heterocycles to be among pharmaceutically active and interesting compounds (Isloor *et al.*, 2009; Vijesh *et al.*, 2011) which justifies our continuing efforts in designing novel heterocyclic molecules of biological importance and study their respective molecular and crystal structure.

The title compound is a derivative of 1,4-dihydroquinoline and does not adopt its aromatic tautomeric form as a quinoline derivative. There are two independent molecules in the asymmetric unit (Fig. 1).

In the crystal, classical N—H···O hydrogen bonds (Table 1) link the alternating independent molecules into chains in [-110] (Fig. 2). In the chain, the quinoline planes of independent molecules are almost perpendicular to each other forming a dihedral angle of 89.8 (1)°. The π–π interactions between the aromatic rings of the quinoline bicycles related by inversion centres [for two independent centrosymmetric dimers the shortest intercentroid distances are 3.495 (1) and 3.603 (1) Å, respectively] link hydrogen-bonded chains into layers parallel to the (110) plane. Weak intermolecular C–H···F contacts are observed next to intermolecular C–H···O contacts (Table 1). In every case, the range of these contacts falls by more than 0.1 Å below the sum of van-der-Waals radii of the atoms participating in them. While the C–H···O contacts stem from one of the hydrogen atoms on the phenyl moiety bearing the trifluoromethyl substituent and apply the ethereal oxygen atom as acceptor, the classical hydrogen bonds invariably have double bonded oxygen atoms as acceptors. These hydrogen bonds intermittently connect the two different molecules present in the asymmetric unit into chains along [-110] and show bifurcation between the two double bonded oxygen atoms in one case. In total, these contacts connect the molecules to planes parallel to *ab*. In terms of graph-set analysis (Etter *et al.*, 1990; Bernstein *et al.*, 1995), the descriptor for the classical hydrogen bonds is *DDD* on the unary level (taking into account the bifurcation). The descriptor for the C–H···O contacts is *D* while a *C*^1^_1_(11) descriptor is found for the C–H···F contacts on the same level.

### Experimental

Diethyl({[3-(trifluoromethyl)phenyl]amino}methylene)malonate (10.0 g, 0.030 mol) and Dowtherm (100 ml) were heated to 250 °C for 5 h. The reaction mixture was then cooled to 25 °C and stirred in *n*-hexane (150 ml) for 10 min. The solid product obtained was filtered, dried and recrystallized from ethanol, yield: 8.0 g (93.0%) (Thomas *et al.*, 2011).

### Refinement

C-bound H atoms were placed in calculated positions (C–H 0.95 Å for aromatic and vinylic carbon atoms and C–H 0.99 Å for methylene groups) and were included in the refinement in the riding model approximation, with *U*(H) set to 1.2*U*_eq_(C). The H atoms of the methyl groups were allowed to rotate with a fixed angle around the C–C bond to best fit the experimental electron density (HFIX 137 in the *SHELX* program suite (Sheldrick, 2008)), with *U*(H) set to 1.5*U*_eq_(C). Both nitrogen-bound H atoms were located on a difference Fourier map and refined freely.

### Crystal data

**Table:**

C_13_H_10_F_3_NO_3_	*Z* = 4
*M**_r_* = 285.22	*F*(000) = 584
Triclinic, *P*1	*D*_x_ = 1.577 Mg m^−^^3^
Hall symbol: -P 1	Melting point = 570–568 K
*a* = 9.8248 (3) Å	Mo *K*α radiation, λ = 0.71073 Å
*b* = 11.0222 (3) Å	Cell parameters from 9882 reflections
*c* = 12.3450 (4) Å	θ = 2.5–28.3°
α = 72.934 (1)°	µ = 0.14 mm^−^^1^
β = 74.167 (1)°	*T* = 200 K
γ = 74.059 (1)°	Platelet, colourless
*V* = 1201.67 (6) Å^3^	0.53 × 0.38 × 0.32 mm

### Data collection

**Table:**

Bruker APEXII CCD diffractometer	5963 independent reflections
Radiation source: fine-focus sealed tube	5051 reflections with *I* > 2σ(*I*)
Graphite monochromator	*R*_int_ = 0.016
φ and ω scans	θ_max_ = 28.3°, θ_min_ = 1.8°
Absorption correction: multi-scan (*SADABS*; Bruker, 2008)	*h* = −13→13
*T*_min_ = 0.928, *T*_max_ = 0.956	*k* = −14→14
21419 measured reflections	*l* = −16→16

### Refinement

**Table:**

Refinement on *F*^2^	Primary atom site location: structure-invariant direct methods
Least-squares matrix: full	Secondary atom site location: difference Fourier map
*R*[*F*^2^ > 2σ(*F*^2^)] = 0.040	Hydrogen site location: inferred from neighbouring sites
*wR*(*F*^2^) = 0.116	H atoms treated by a mixture of independent and constrained refinement
*S* = 1.04	*w* = 1/[σ^2^(*F*_o_^2^) + (0.0593*P*)^2^ + 0.3846*P*] where *P* = (*F*_o_^2^ + 2*F*_c_^2^)/3
5963 reflections	(Δ/σ)_max_ < 0.001
371 parameters	Δρ_max_ = 0.39 e Å^−^^3^
0 restraints	Δρ_min_ = −0.23 e Å^−^^3^

### Fractional atomic coordinates and isotropic or equivalent isotropic displacement parameters (Å^2^)

**Table:**

	*x*	*y*	*z*	*U*_iso_*/*U*_eq_	
F11	0.33975 (9)	0.25175 (8)	0.03791 (8)	0.0439 (2)	
F12	0.44442 (10)	0.35929 (10)	0.09612 (10)	0.0568 (3)	
F13	0.47655 (10)	0.37218 (10)	−0.08575 (9)	0.0631 (3)	
F21	0.16132 (9)	0.25934 (8)	0.55061 (8)	0.0470 (2)	
F22	0.05981 (10)	0.16771 (11)	0.71858 (8)	0.0581 (3)	
F23	0.01521 (11)	0.13966 (11)	0.56807 (11)	0.0657 (3)	
O11	−0.20937 (11)	0.70312 (9)	0.17938 (8)	0.0405 (2)	
O12	−0.33287 (11)	0.58234 (11)	0.40381 (10)	0.0474 (3)	
O13	−0.19356 (10)	0.39636 (9)	0.48120 (8)	0.0360 (2)	
O21	0.71392 (13)	−0.18698 (10)	0.68194 (9)	0.0488 (3)	
O22	0.83982 (11)	−0.07326 (11)	0.79574 (11)	0.0533 (3)	
O23	0.69974 (11)	0.11050 (11)	0.84210 (9)	0.0445 (2)	
N1	0.12165 (11)	0.38724 (9)	0.19918 (9)	0.0270 (2)	
H1	0.190 (2)	0.3151 (18)	0.2098 (16)	0.046 (5)*	
N2	0.38130 (11)	0.12577 (10)	0.69417 (9)	0.0276 (2)	
H2	0.3130 (19)	0.1915 (17)	0.7026 (15)	0.039 (4)*	
C101	0.13071 (12)	0.48491 (11)	0.09948 (10)	0.0248 (2)	
C102	0.25053 (13)	0.47904 (12)	0.00530 (11)	0.0291 (2)	
C103	0.25240 (14)	0.57880 (13)	−0.09286 (11)	0.0341 (3)	
H103	0.3331	0.5743	−0.1560	0.041*	
C104	0.13755 (15)	0.68645 (13)	−0.10101 (11)	0.0345 (3)	
H104	0.1399	0.7543	−0.1695	0.041*	
C105	0.02133 (14)	0.69401 (11)	−0.00978 (11)	0.0307 (2)	
H105	−0.0566	0.7675	−0.0154	0.037*	
C106	0.01622 (12)	0.59419 (11)	0.09183 (10)	0.0251 (2)	
C107	0.37692 (14)	0.36615 (14)	0.01239 (12)	0.0372 (3)	
C108	−0.11013 (12)	0.60598 (11)	0.18858 (10)	0.0269 (2)	
C109	−0.10586 (12)	0.49925 (11)	0.28998 (10)	0.0255 (2)	
C110	0.00998 (12)	0.39563 (11)	0.28906 (10)	0.0261 (2)	
H110	0.0107	0.3260	0.3562	0.031*	
C111	−0.22344 (12)	0.50028 (11)	0.39440 (10)	0.0281 (2)	
C112	−0.29520 (15)	0.38902 (15)	0.59208 (11)	0.0383 (3)	
H11A	−0.2959	0.2970	0.6318	0.046*	
H11B	−0.3939	0.4329	0.5794	0.046*	
C113	−0.25346 (18)	0.45247 (16)	0.66662 (13)	0.0459 (3)	
H11C	−0.1546	0.4106	0.6770	0.069*	
H11D	−0.3202	0.4435	0.7423	0.069*	
H11E	−0.2583	0.5448	0.6292	0.069*	
C201	0.36760 (13)	0.02791 (11)	0.65228 (10)	0.0278 (2)	
C202	0.24340 (14)	0.03303 (13)	0.61281 (11)	0.0333 (3)	
C203	0.23449 (18)	−0.07003 (15)	0.57567 (13)	0.0445 (3)	
H203	0.1504	−0.0669	0.5502	0.053*	
C204	0.3472 (2)	−0.17898 (15)	0.57496 (14)	0.0498 (4)	
H204	0.3396	−0.2497	0.5494	0.060*	
C205	0.46903 (18)	−0.18417 (13)	0.61103 (12)	0.0423 (3)	
H205	0.5461	−0.2582	0.6093	0.051*	
C206	0.48144 (14)	−0.08153 (11)	0.65054 (10)	0.0309 (3)	
C207	0.12056 (14)	0.14904 (15)	0.61157 (12)	0.0390 (3)	
C208	0.61343 (14)	−0.09151 (12)	0.69029 (10)	0.0322 (3)	
C209	0.61215 (13)	0.01330 (12)	0.73845 (10)	0.0295 (2)	
C210	0.49545 (13)	0.11609 (11)	0.73754 (10)	0.0277 (2)	
H210	0.4959	0.1844	0.7697	0.033*	
C211	0.73085 (13)	0.00904 (13)	0.79296 (11)	0.0341 (3)	
C212	0.79490 (17)	0.11036 (17)	0.91340 (13)	0.0465 (4)	
H21A	0.8003	0.2007	0.9067	0.056*	
H21B	0.8936	0.0623	0.8857	0.056*	
C213	0.74080 (19)	0.04817 (17)	1.03738 (13)	0.0484 (4)	
H21C	0.6426	0.0950	1.0643	0.073*	
H21D	0.8048	0.0511	1.0846	0.073*	
H21E	0.7395	−0.0424	1.0444	0.073*	

### Atomic displacement parameters (Å^2^)

**Table:**

	*U*^11^	*U*^22^	*U*^33^	*U*^12^	*U*^13^	*U*^23^
F11	0.0417 (4)	0.0334 (4)	0.0518 (5)	−0.0007 (3)	−0.0053 (4)	−0.0137 (4)
F12	0.0366 (4)	0.0627 (6)	0.0764 (7)	0.0050 (4)	−0.0282 (5)	−0.0237 (5)
F13	0.0424 (5)	0.0589 (6)	0.0598 (6)	0.0010 (4)	0.0196 (4)	−0.0110 (5)
F21	0.0428 (5)	0.0386 (4)	0.0502 (5)	−0.0027 (3)	−0.0098 (4)	−0.0021 (4)
F22	0.0385 (5)	0.0789 (7)	0.0411 (5)	0.0022 (4)	0.0011 (4)	−0.0130 (5)
F23	0.0512 (6)	0.0676 (7)	0.0894 (8)	−0.0103 (5)	−0.0422 (6)	−0.0120 (6)
O11	0.0428 (5)	0.0338 (5)	0.0311 (5)	0.0145 (4)	−0.0083 (4)	−0.0078 (4)
O12	0.0306 (5)	0.0467 (6)	0.0462 (6)	0.0050 (4)	0.0015 (4)	−0.0042 (5)
O13	0.0353 (5)	0.0409 (5)	0.0252 (4)	−0.0031 (4)	−0.0036 (3)	−0.0049 (4)
O21	0.0574 (6)	0.0394 (5)	0.0384 (5)	0.0243 (5)	−0.0201 (5)	−0.0154 (4)
O22	0.0336 (5)	0.0522 (6)	0.0669 (8)	0.0066 (5)	−0.0198 (5)	−0.0091 (5)
O23	0.0421 (5)	0.0527 (6)	0.0424 (6)	−0.0029 (4)	−0.0195 (4)	−0.0136 (5)
N1	0.0258 (5)	0.0247 (5)	0.0271 (5)	0.0029 (4)	−0.0081 (4)	−0.0061 (4)
N2	0.0264 (5)	0.0251 (5)	0.0278 (5)	0.0028 (4)	−0.0066 (4)	−0.0078 (4)
C101	0.0260 (5)	0.0251 (5)	0.0254 (5)	−0.0036 (4)	−0.0083 (4)	−0.0082 (4)
C102	0.0270 (5)	0.0310 (6)	0.0305 (6)	−0.0051 (4)	−0.0058 (4)	−0.0101 (5)
C103	0.0346 (6)	0.0402 (7)	0.0286 (6)	−0.0128 (5)	−0.0019 (5)	−0.0094 (5)
C104	0.0448 (7)	0.0322 (6)	0.0274 (6)	−0.0126 (5)	−0.0099 (5)	−0.0020 (5)
C105	0.0381 (6)	0.0250 (5)	0.0299 (6)	−0.0034 (4)	−0.0130 (5)	−0.0055 (4)
C106	0.0283 (5)	0.0241 (5)	0.0243 (5)	−0.0021 (4)	−0.0094 (4)	−0.0076 (4)
C107	0.0269 (6)	0.0407 (7)	0.0392 (7)	−0.0036 (5)	−0.0005 (5)	−0.0118 (6)
C108	0.0285 (5)	0.0263 (5)	0.0258 (5)	0.0023 (4)	−0.0099 (4)	−0.0094 (4)
C109	0.0253 (5)	0.0264 (5)	0.0251 (5)	−0.0016 (4)	−0.0079 (4)	−0.0079 (4)
C110	0.0274 (5)	0.0252 (5)	0.0248 (5)	−0.0017 (4)	−0.0090 (4)	−0.0050 (4)
C111	0.0260 (5)	0.0299 (5)	0.0291 (6)	−0.0049 (4)	−0.0063 (4)	−0.0086 (4)
C112	0.0366 (7)	0.0491 (8)	0.0279 (6)	−0.0163 (6)	−0.0004 (5)	−0.0061 (5)
C113	0.0509 (8)	0.0551 (9)	0.0326 (7)	−0.0138 (7)	−0.0049 (6)	−0.0128 (6)
C201	0.0341 (6)	0.0260 (5)	0.0197 (5)	−0.0044 (4)	−0.0050 (4)	−0.0026 (4)
C202	0.0383 (6)	0.0350 (6)	0.0250 (6)	−0.0102 (5)	−0.0082 (5)	−0.0010 (5)
C203	0.0595 (9)	0.0459 (8)	0.0352 (7)	−0.0221 (7)	−0.0159 (6)	−0.0045 (6)
C204	0.0807 (12)	0.0363 (7)	0.0400 (8)	−0.0201 (7)	−0.0168 (8)	−0.0093 (6)
C205	0.0650 (9)	0.0265 (6)	0.0319 (6)	−0.0031 (6)	−0.0107 (6)	−0.0074 (5)
C206	0.0424 (7)	0.0249 (5)	0.0202 (5)	−0.0009 (5)	−0.0062 (5)	−0.0038 (4)
C207	0.0313 (6)	0.0496 (8)	0.0348 (7)	−0.0095 (6)	−0.0103 (5)	−0.0042 (6)
C208	0.0381 (6)	0.0282 (6)	0.0200 (5)	0.0072 (5)	−0.0064 (4)	−0.0032 (4)
C209	0.0283 (5)	0.0296 (6)	0.0233 (5)	0.0018 (4)	−0.0053 (4)	−0.0029 (4)
C210	0.0279 (5)	0.0269 (5)	0.0247 (5)	−0.0012 (4)	−0.0046 (4)	−0.0057 (4)
C211	0.0295 (6)	0.0372 (6)	0.0282 (6)	−0.0037 (5)	−0.0066 (5)	0.0010 (5)
C212	0.0445 (8)	0.0630 (9)	0.0355 (7)	−0.0223 (7)	−0.0154 (6)	−0.0003 (6)
C213	0.0564 (9)	0.0523 (9)	0.0335 (7)	−0.0136 (7)	−0.0072 (6)	−0.0055 (6)

### Geometric parameters (Å, º)

**Table:**

F11—C107	1.3341 (16)	C108—C109	1.4457 (16)
F12—C107	1.3470 (17)	C109—C110	1.3735 (15)
F13—C107	1.3310 (16)	C109—C111	1.4782 (16)
F21—C207	1.3307 (17)	C110—H110	0.9500
F22—C207	1.3437 (17)	C112—C113	1.498 (2)
F23—C207	1.3285 (16)	C112—H11A	0.9900
O11—C108	1.2351 (14)	C112—H11B	0.9900
O12—C111	1.2029 (15)	C113—H11C	0.9800
O13—C111	1.3464 (15)	C113—H11D	0.9800
O13—C112	1.4529 (15)	C113—H11E	0.9800
O21—C208	1.2370 (15)	C201—C206	1.4027 (16)
O22—C211	1.1994 (16)	C201—C202	1.4141 (17)
O23—C211	1.3495 (18)	C202—C203	1.375 (2)
O23—C212	1.4479 (17)	C202—C207	1.4976 (19)
N1—C110	1.3355 (15)	C203—C204	1.393 (2)
N1—C101	1.3773 (15)	C203—H203	0.9500
N1—H1	0.892 (19)	C204—C205	1.368 (2)
N2—C210	1.3358 (15)	C204—H204	0.9500
N2—C201	1.3763 (15)	C205—C206	1.4017 (18)
N2—H2	0.851 (18)	C205—H205	0.9500
C101—C106	1.4043 (15)	C206—C208	1.4737 (18)
C101—C102	1.4132 (16)	C208—C209	1.4420 (18)
C102—C103	1.3765 (18)	C209—C210	1.3727 (15)
C102—C107	1.4981 (17)	C209—C211	1.4815 (17)
C103—C104	1.3956 (19)	C210—H210	0.9500
C103—H103	0.9500	C212—C213	1.495 (2)
C104—C105	1.3712 (18)	C212—H21A	0.9900
C104—H104	0.9500	C212—H21B	0.9900
C105—C106	1.4044 (16)	C213—H21C	0.9800
C105—H105	0.9500	C213—H21D	0.9800
C106—C108	1.4757 (16)	C213—H21E	0.9800
			
C111—O13—C112	117.59 (10)	H11C—C113—H11D	109.5
C211—O23—C212	117.55 (12)	C112—C113—H11E	109.5
C110—N1—C101	121.95 (10)	H11C—C113—H11E	109.5
C110—N1—H1	114.9 (12)	H11D—C113—H11E	109.5
C101—N1—H1	123.2 (12)	N2—C201—C206	118.05 (11)
C210—N2—C201	121.93 (10)	N2—C201—C202	122.66 (11)
C210—N2—H2	115.4 (11)	C206—C201—C202	119.28 (11)
C201—N2—H2	122.2 (11)	C203—C202—C201	119.74 (13)
N1—C101—C106	118.41 (10)	C203—C202—C207	119.36 (13)
N1—C101—C102	122.48 (10)	C201—C202—C207	120.90 (11)
C106—C101—C102	119.12 (10)	C202—C203—C204	120.80 (14)
C103—C102—C101	119.82 (11)	C202—C203—H203	119.6
C103—C102—C107	119.71 (11)	C204—C203—H203	119.6
C101—C102—C107	120.46 (11)	C205—C204—C203	120.02 (13)
C102—C103—C104	121.00 (12)	C205—C204—H204	120.0
C102—C103—H103	119.5	C203—C204—H204	120.0
C104—C103—H103	119.5	C204—C205—C206	120.81 (13)
C105—C104—C103	119.78 (11)	C204—C205—H205	119.6
C105—C104—H104	120.1	C206—C205—H205	119.6
C103—C104—H104	120.1	C205—C206—C201	119.33 (13)
C104—C105—C106	120.70 (11)	C205—C206—C208	119.22 (12)
C104—C105—H105	119.7	C201—C206—C208	121.44 (11)
C106—C105—H105	119.7	F23—C207—F21	106.70 (11)
C101—C106—C105	119.58 (11)	F23—C207—F22	106.69 (12)
C101—C106—C108	121.19 (10)	F21—C207—F22	105.13 (12)
C105—C106—C108	119.22 (10)	F23—C207—C202	112.96 (13)
F13—C107—F11	106.66 (11)	F21—C207—C202	113.00 (11)
F13—C107—F12	106.61 (11)	F22—C207—C202	111.81 (11)
F11—C107—F12	105.45 (12)	O21—C208—C209	125.29 (13)
F13—C107—C102	112.78 (12)	O21—C208—C206	119.16 (12)
F11—C107—C102	113.23 (10)	C209—C208—C206	115.54 (10)
F12—C107—C102	111.58 (11)	C210—C209—C208	118.91 (11)
O11—C108—C109	124.72 (11)	C210—C209—C211	119.79 (11)
O11—C108—C106	119.80 (11)	C208—C209—C211	121.25 (11)
C109—C108—C106	115.48 (10)	N2—C210—C209	123.90 (11)
C110—C109—C108	119.21 (10)	N2—C210—H210	118.0
C110—C109—C111	119.66 (10)	C209—C210—H210	118.0
C108—C109—C111	121.13 (10)	O22—C211—O23	123.44 (13)
N1—C110—C109	123.71 (10)	O22—C211—C209	125.73 (13)
N1—C110—H110	118.1	O23—C211—C209	110.82 (10)
C109—C110—H110	118.1	O23—C212—C213	110.57 (12)
O12—C111—O13	122.73 (11)	O23—C212—H21A	109.5
O12—C111—C109	125.78 (11)	C213—C212—H21A	109.5
O13—C111—C109	111.49 (10)	O23—C212—H21B	109.5
O13—C112—C113	110.26 (11)	C213—C212—H21B	109.5
O13—C112—H11A	109.6	H21A—C212—H21B	108.1
C113—C112—H11A	109.6	C212—C213—H21C	109.5
O13—C112—H11B	109.6	C212—C213—H21D	109.5
C113—C112—H11B	109.6	H21C—C213—H21D	109.5
H11A—C112—H11B	108.1	C212—C213—H21E	109.5
C112—C113—H11C	109.5	H21C—C213—H21E	109.5
C112—C113—H11D	109.5	H21D—C213—H21E	109.5
			
C110—N1—C101—C106	−2.05 (16)	C210—N2—C201—C206	−3.23 (17)
C110—N1—C101—C102	177.88 (10)	C210—N2—C201—C202	175.92 (11)
N1—C101—C102—C103	178.98 (11)	N2—C201—C202—C203	−177.79 (12)
C106—C101—C102—C103	−1.09 (17)	C206—C201—C202—C203	1.35 (18)
N1—C101—C102—C107	−2.20 (17)	N2—C201—C202—C207	1.81 (18)
C106—C101—C102—C107	177.73 (11)	C206—C201—C202—C207	−179.05 (11)
C101—C102—C103—C104	0.15 (18)	C201—C202—C203—C204	−0.8 (2)
C107—C102—C103—C104	−178.67 (12)	C207—C202—C203—C204	179.55 (13)
C102—C103—C104—C105	0.53 (19)	C202—C203—C204—C205	−0.3 (2)
C103—C104—C105—C106	−0.26 (19)	C203—C204—C205—C206	0.9 (2)
N1—C101—C106—C105	−178.71 (10)	C204—C205—C206—C201	−0.4 (2)
C102—C101—C106—C105	1.35 (16)	C204—C205—C206—C208	179.11 (13)
N1—C101—C106—C108	0.99 (16)	N2—C201—C206—C205	178.44 (11)
C102—C101—C106—C108	−178.94 (10)	C202—C201—C206—C205	−0.74 (18)
C104—C105—C106—C101	−0.69 (17)	N2—C201—C206—C208	−1.05 (17)
C104—C105—C106—C108	179.60 (11)	C202—C201—C206—C208	179.77 (11)
C103—C102—C107—F13	−4.70 (18)	C203—C202—C207—F23	−2.68 (18)
C101—C102—C107—F13	176.48 (11)	C201—C202—C207—F23	177.71 (12)
C103—C102—C107—F11	−125.94 (13)	C203—C202—C207—F21	−123.94 (14)
C101—C102—C107—F11	55.24 (16)	C201—C202—C207—F21	56.46 (16)
C103—C102—C107—F12	115.27 (14)	C203—C202—C207—F22	117.68 (14)
C101—C102—C107—F12	−63.55 (15)	C201—C202—C207—F22	−61.92 (16)
C101—C106—C108—O11	−179.90 (11)	C205—C206—C208—O21	3.99 (18)
C105—C106—C108—O11	−0.19 (17)	C201—C206—C208—O21	−176.53 (12)
C101—C106—C108—C109	0.70 (16)	C205—C206—C208—C209	−175.03 (11)
C105—C106—C108—C109	−179.60 (10)	C201—C206—C208—C209	4.46 (17)
O11—C108—C109—C110	179.19 (12)	O21—C208—C209—C210	177.19 (12)
C106—C108—C109—C110	−1.43 (15)	C206—C208—C209—C210	−3.86 (16)
O11—C108—C109—C111	−1.12 (18)	O21—C208—C209—C211	−5.5 (2)
C106—C108—C109—C111	178.25 (10)	C206—C208—C209—C211	173.49 (11)
C101—N1—C110—C109	1.34 (17)	C201—N2—C210—C209	3.95 (18)
C108—C109—C110—N1	0.50 (17)	C208—C209—C210—N2	−0.11 (18)
C111—C109—C110—N1	−179.19 (10)	C211—C209—C210—N2	−177.50 (11)
C112—O13—C111—O12	−3.68 (18)	C212—O23—C211—O22	−7.8 (2)
C112—O13—C111—C109	175.91 (10)	C212—O23—C211—C209	171.24 (11)
C110—C109—C111—O12	−176.54 (12)	C210—C209—C211—O22	−177.47 (13)
C108—C109—C111—O12	3.78 (19)	C208—C209—C211—O22	5.2 (2)
C110—C109—C111—O13	3.89 (15)	C210—C209—C211—O23	3.55 (16)
C108—C109—C111—O13	−175.80 (10)	C208—C209—C211—O23	−173.77 (11)
C111—O13—C112—C113	−90.46 (14)	C211—O23—C212—C213	−91.90 (16)

### Hydrogen-bond geometry (Å, º)

**Table:**

*D*—H···*A*	*D*—H	H···*A*	*D*···*A*	*D*—H···*A*
N1—H1···O21^i^	0.892 (19)	1.875 (19)	2.6588 (13)	145.5 (16)
N2—H2···O11^ii^	0.851 (18)	2.011 (17)	2.7178 (13)	139.9 (16)
N2—H2···O12^ii^	0.851 (18)	2.487 (17)	3.0380 (15)	123.2 (14)
C212—H21*B*···F22^iii^	0.99	2.46	3.0909 (18)	121
C204—H204···O13^iv^	0.95	2.60	3.4691 (18)	153

Symmetry codes: (i) −*x*+1, −*y*, −*z*+1; (ii) −*x*, −*y*+1, −*z*+1; (iii) *x*+1, *y*, *z*; (iv) −*x*, −*y*, −*z*+1.
